# Pomolic Acid Ameliorates Fibroblast Activation and Renal Interstitial Fibrosis through Inhibition of SMAD-STAT Signaling Pathways

**DOI:** 10.3390/molecules23092236

**Published:** 2018-09-03

**Authors:** Ji-Hyun Park, Kyung Mi Jang, Hyun Jin An, Jung-Yeon Kim, Mi-Gyeong Gwon, Hyemin Gu, Byoungduck Park, Kwan-Kyu Park

**Affiliations:** 1 Department of Pathology, College of Medicine, Catholic University of Daegu, Daegu 42472, Korea; jihyunp@cu.ac.kr (J.-H.P.); ahj119@cu.ac.kr (H.J.A.); jy1118@cu.ac.kr (J.-Y.K.); daldy88@cu.ac.kr (M.-G.G.); guhm1207@cu.ac.kr (H.G.); 2 Department of Paediatrics, College of Medicine, Yeungnam University, Daegu 42415, Korea; fortune001j@gmail.com; 3 College of Pharmacy, Keimyung University, Daegu 42601, Korea; bpark@kmu.ac.kr

**Keywords:** TGF-β1, Pomolic acid, Renal fibrosis, fibroblast, ECM

## Abstract

Fibrosis is a common pathological feature in most kinds of chronic kidney disease. Transforming growth factor β1 (TGF-β1) signaling is the master pathway regulating kidney fibrosis pathogenesis, in which mothers against decapentaplegic homolog 3 (SMAD3) with signal transducer and activator of transcription 3 (STAT3) act as the integrator of various pro-fibrosis signals. We examine the effects of pomolic acid (PA) on mice with unilateral ureteral obstruction (UUO) and TGF-β1 stimulated kidney fibroblast cells. UUO mice were observed severe tubular atrophy, and tubulointerstitial fibrosis and extracellular matrix (ECM) deposition at seven days postoperatively. However, PA-treated UUO mice demonstrated only moderate injury, minimal fibrosis, and larger reductions in the expression of ECM protein and epithelial-mesenchymal transition (EMT) progress. PA inhibited the SMAD-STAT phosphorylation in UUO mice. PA effects were also confirmed in TGF-β1 stimulated kidney fibroblast cells. In this study, we first demonstrated that PA ameliorates fibroblast activation and renal interstitial fibrosis. Our results indicate that PA may be useful as a potential candidate in the prevention of chronic kidney disease.

## 1. Introduction

Renal interstitial fibrosis is the destruction of renal parenchyma and the progressive loss of kidney function to end-stage renal disease and is characterized by fibroblast activation and the excessive production and deposition of extracellular matrix (ECM) [1]. A key step in the evolution of chronic kidney disease is the transformation of renal fibroblasts to α-smooth muscle actin (SMA) positive myofibroblasts [2]. These activated fibroblasts are the cells that are principally responsible for ECM production, and their activation is regarded as a significant event in the pathogenesis of renal fibrosis [3]. The progression of renal disease in UUO (unilateral ureteral obstruction) mice is associated with epithelial-to-mesenchymal transition (EMT) in which there is reciprocal upregulation of α-SMA expression and decrea in E-cadherin expression [4]. With the loss of epithelial cell properties, myofibroblasts proliferate, migrate, and produce and deposit large amounts of ECM in the renal interstitium [5]. However, the molecular mechanisms underlying fibroblast activation are not fully understood.

Transforming growth factor β1 (TGF-β1) plays a central role in the pathogenesis of renal fibrosis through the activation of a cascade of intracellular signaling pathways [6]. TGF-β1 signaling is among the most intensively studied causes of fibrosis, employing SMAD and non-SMAD pathways that induce the gene expression needed for resting fibroblast activation to myofibroblasts [7]. Among the signaling pathways associated with renal tubulointerstitial injury, the SMAD and non-SMAD mediated TGF-β1 pathway occupies a crucial position in this process [8]. TGF-β1 is markedly upregulated and SMAD and STAT is highly activated in the fibrotic kidney [2,9]. Evidence suggests that activation of the SMAD signaling cascade is important in the regulation of ECM protein expression and tissue fibrosis [6,10]. Furthermore, STAT3 activation mediates the stimulation of renal interstitial fibroblasts and the progression of renal fibrosis in UUO models [11]. Thus, inhibition of SMAD and STAT signaling is important in renal fibrosis.

Pomolic acid (PA) is a pentacyclic triterpene isolated from *Euscaphis japonica* (Tunb.) Kantiz (Staphyleaceae) which is found in China, Japan, and Korea [12]. A previous study reported that PA exhibited a protective effect against hepatic stellate cells [13] and it has also demonstrated antiproliferative activity against human gastric adenocarcinoma, human uterine carcinoma, and murine melanoma [14]. Additionally, we previously demonstrated that PA inhibits the invasion of breast cancer cells through the NF-κB, MAPK, and mTOR signaling pathways [15,16]. However, the molecular mechanisms of the anti-fibrotic potential of PA in renal fibrosis have not yet been elucidated.

The present study was established to test the possible renoprotective effect of PA through its activation of inhibitory fibroblasts in obstructive nephropathy. We found that PA suppresses fibroblast activation by affecting multiple TGF-β1-mediated molecules involved in kidney injury.

## 2. Results

### 2.1. PA Improves Histopathological Changes in UUO Mice

**Figure 1:**
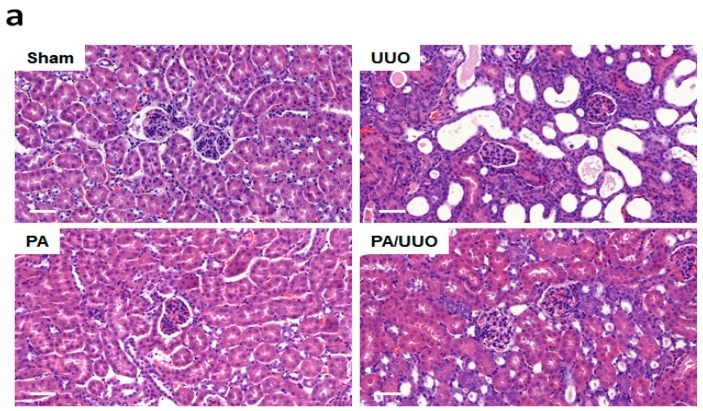

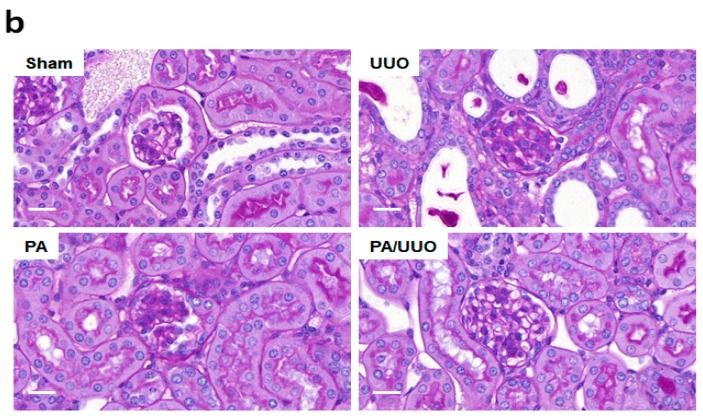
The effects of pomolic acid (PA) on histological alterations in unilateral ureteral obstruction (UUO) mice. Histopathological alterations in the hematoxylin and eosin (H&E)-stained (**a**) and the periodic acid–Schiff (PAS)-stained slides (**b**). H&E: Scale bar 50 μm. PAS: Scale bar 20 μm.

We investigated the effects of pomolic acid (PA) in the renal interstitial fibrosis using UUO mice. Hematoxylin and eosin (H&E) staining observed normal renal cortex in the Sham and PA groups (Figure 1a). In the UUO group, interstitial inflammatory cell infiltration, swollen epithelial cells, partial tubular expansion, and severe tubular atrophy cells were observed. These features of UUO group was diluted strongly in PA/UUO group. In the glomerulus of the UUO group, extensive mesangial matrix expansion was observed by PAS staining. The PA/UUO group significantly decreased the mesangial area (Figure 1b).

**Figure 2:**
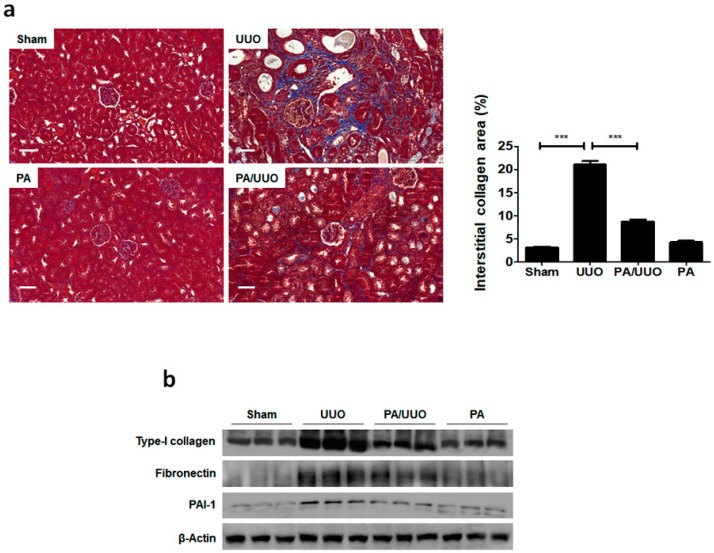

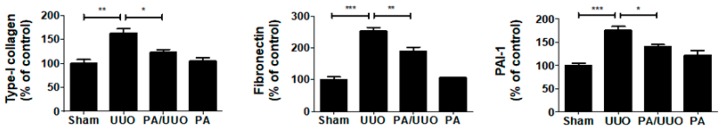
PA suppresses collagen and extracellular matrix (ECM) accumulation in UUO mice. (**a**) The kidney sections are stained with Masson’s trichrome, which accentuates interstitial fibrosis by staining the collagen blue. Scale bar 50 μm. The semi-quantitative analysis of collagen blue areas of the obstructed kidney in each group. These are representative images from each study group. (**b**) Immunoblot results show the effects of PA on ECM accumulation in UUO mice. β-Actin was used to confirm equal sample loading. The data are representative of three independent experiments and quantified as mean values ± SEM. Tukey’s multiple comparison test, * *p* < 0.05, ** *p* < 0.01, *** *p* < 0.001 compared to control.

Consistent with the changes in the glomerulus, Masson’s trichrome staining showed that the PA/UUO group exhibited a marked reduction from the increased collagen deposition levels observed in the UUO group (Figure 2a). A similar suppressive effect on collagen and ECM deposition was confirmed by immunoblot analysis (Figure 2b). The PA/UUO group exhibited strongly attenuated type-I collagen, fibronectin, and PAI-1 expression as compared to the UUO group. This indicates that PA may attenuate obstructive nephropathy in vivo.

### 2.2. PA Attenuates UUO-Induced EMT Progression

**Figure 3:**
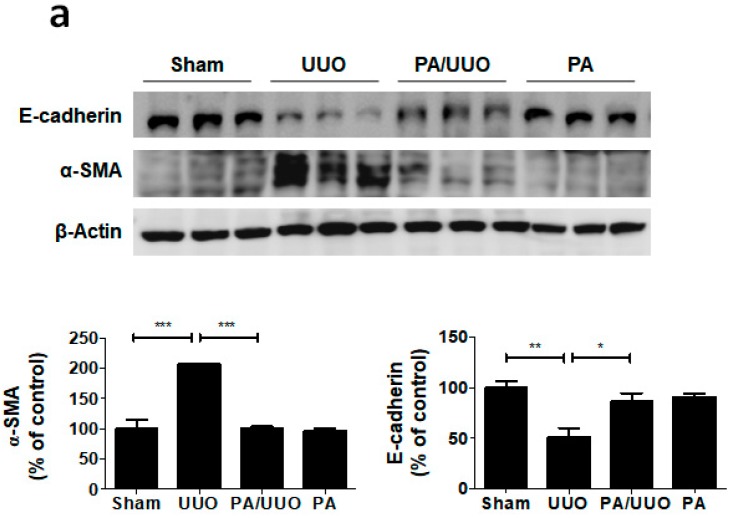

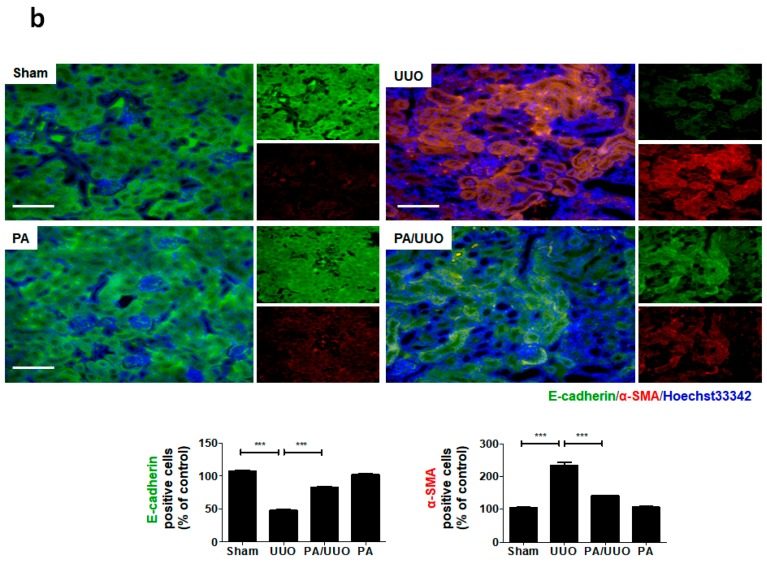
Effect of PA on E-cadherin and α-SMA expression in UUO mice. (**a**) Immunoblot results show the effects of PA on the inhibition of UUO-induced changes in EMT markers, including E-cadherin and α-SMA. β-Actin was used to confirm equal sample loading. (**b**) Immunofluorescence double staining for E-cadherin (green) and α-SMA (red) localization. Cells were counterstained with Hoechst 33342 (blue). Scale bar 100 μm. The data are representative of three independent experiments and quantified as mean values ± SEM. Tukey’s multiple comparison test, * *p* < 0.05, ** *p* < 0.01, *** *p* < 0.001 compared to control.

A key feature of renal fibrosis, EMT is characterized by the loss of intracellular epithelial adhesion molecules (E-cadherin) and the generation of mesenchymal phenotypes (α-SMA) [16]. As shown in Figure 3a, the UUO group exhibited downregulation of E-cadherin and upregulation of α-SMA occurred, whereas the PA/UUO group exhibited increasing resistance to the progression of EMT. This resistance to the progression of EMT was also confirmed by immunofluorescence staining. The Sham and PA group observed expression of E-cadherin localized at the cell border and low levels of α-SMA expression (Figure 3b). The UUO group exhibited a loss of E-cadherin accompanied by an increased α-SMA expression. The PA/UUO group observed resistance of UUO-mediated EMT progression.

### 2.3. PA Inhibits TGF-β1 Stimulated Fibroblast Activation

**Figure 4:**
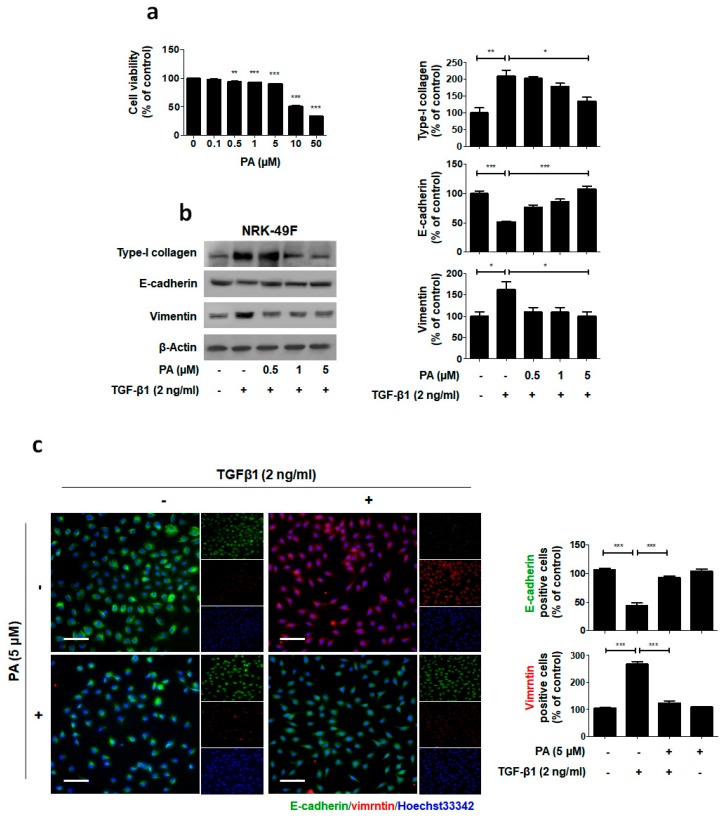
Suppression of TGF-β1-induced ECM and EMT by PA. (**a**) Cells were treated with PA for 24 h, and then MTT assays were conducted. (**b**) Immunoblot results show the effect of PA on the inhibition of TGF-β1-induced type-I collagen expression and changes in EMT markers, including E-cadherin and vimentin. β-Actin was used to confirm equal sample loading. (**c**) Immunofluorescence double staining for E-cadherin (green) and vimentin (red) localization. Cells were counterstained with Hoechst 33342 (blue). Scale bar 50 μm. The data are representative of three independent experiments and quantified as mean values ± SEM. Tukey’s multiple comparison test, * *p* < 0.05, ** *p* < 0.01, *** *p* < 0.001 compared to control.

The above data demonstrates that PA inhibited obstructive nephropathy in vivo. To further investigate the role of PA in kidney fibroblast activation, we administered PA to a TGF-β1-treated rat interstitial fibroblast cell line (NRK-49F). First, to determine the cytotoxic effect of PA on the activation of NRK-49F cells, we treated them with PA for 24 h and then conducted MTT assays. We found that PA exhibited mild growth inhibitory activity with a 10% decrease in cell proliferation at 5 μM (Figure 4a). Subsequent experiments were performed using non-toxic PA concentrations of 0.5, 1 and 5 μM. As shown in Figure 4b, the TGF-β1 treatment stimulated type-I collagen expression. In addition, downregulation of E-cadherin and upregulation of vimentin occurred. The PA abrogated the TGF-β1-mediated upregulation of type-I collagen and fibronectin, and cellular resistance to the expression of EMT markers, in a dose-dependent manner.

We also performed immunofluorescence staining to examine the expression of E-cadherin and vimentin in the NRK-49F cells (Figure 4c). PA maintained high localized expression of E-cadherin and showed no increase in vimentin levels in TGF-β1 treated NRK-49F cells. These results demonstrate that PA may elicit its antifibrotic effect by suppressing the fibroblast activation by TGF-β1.

### 2.4. PA Inhibits TGF-β1 Induced Canonical and Non-Canonical Signaling

To suppress the expression of TGF-β1 in fibrogenesis, a strategy has been proposed to block of signaling [17]. Recently our reported that SMADs are important intracellular mediators for TGF-β1 induced responses through their regulation of the transcription of target genes [18,19]. Elsewhere, the selective inhibitor STAT3 inhibited activating interstitial fibroblasts in an obstructive nephropathy model [20].

**Figure 5:**
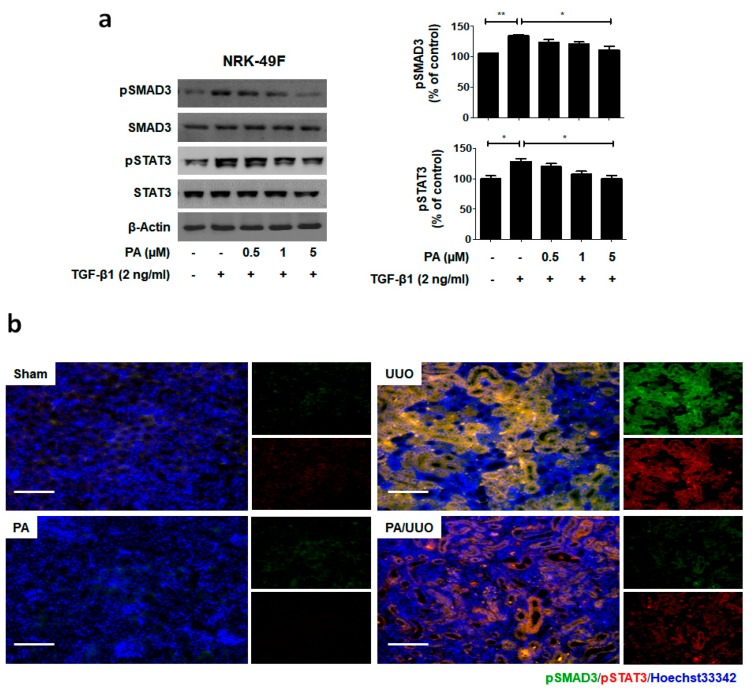

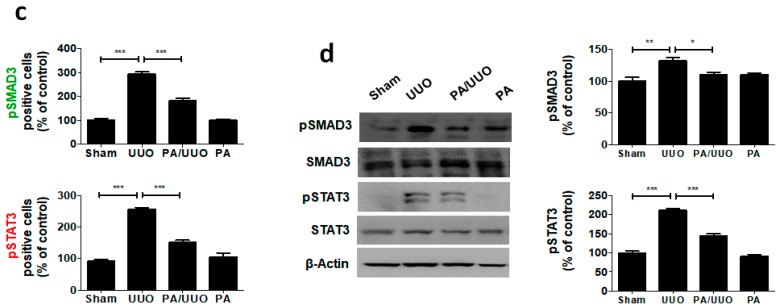
PA inhibits SMAD3-STAT3 signaling in vitro and in vivo. (**a**) Immunoblot shows the effects of PA on the inhibition of phosphorylated SMAD3-STAT3. (**b**) Immunofluorescence double staining for pSMAD3 (green) and pSTAT3 (red) localization. Cells were counterstained with Hoechst 33342 (blue). Scale bar 100 μm. (**c**) Quantification of immunofluorescence double staining. (**d**) Immunoblot results the effects of PA on the SMAD3-STAT3 signaling in UUO mice. β-Actin was used to confirm equal sample loading. The data are representative of three independent experiments and quantified as mean values ± SEM. Tukey’s multiple comparison test, * *p* < 0.05, ** *p* < 0.01, *** *p* < 0.001 compared to control.

To elucidate the molecular mechanism underlying the action of PA within TGF-β1 treatment, we investigated whether it alters the phosphorylation of the SMAD3-STAT3 signaling involved in renal fibrosis. As shown in Figure 5a, PA significantly inhibited TGF-β1 induced phosphorylation of SMAD3 and STAT3 in a dose-dependent manner.

To clarify the molecular mechanism underlying the action of PA in our UUO model, we investigated whether it altered the SMAD3-STAT3 signaling involved in renal fibrosis. The PA/UUO group exhibited significantly suppressed expression of pSMAD3 and pSTAT3 compared to the UUO group (Figure 5b). This similar suppressive effect of PA on pSMAD3 and pSTAT3 expression was confirmed using immunoblotting (Figure 5c). These results demonstrate that PA efficiently downregulates SMAD and STAT activation, resulting in a lasting reduction of phosphorylation in obstructive nephropathy.

## 3. Discussion

Renal fibrosis is a common pathological consequence of chronic kidney disease with tissue fibrosis closely associated with chronic inflammation in numerous pathologies [21]. The UUO model is a representative animal model of obstructive nephropathy that is characterized by progressive tubular-interstitial fibrosis [22]. This model provides the opportunity to investigate disease-specific mechanisms and molecular pathogenesis, and to assess potential novel therapies. In our previous study, UUO was recognized as an established model of progressive tubulointerstitial fibrosis associated with chronic kidney disease of various etiologies [1,23].

Previous studies reported that PA has been shown to have anti-cancer activities, and act against hepatic stellate cells activation, neuroprotective, antioxidant effects, anti-inflammatory and anti-proliferative activity [24,25,26]. Recently, we demonstrated that PA suppresses angiogenesis and invasion of breast cancer cells by mammalian target of rapamycin (mTOR) inhibition [15,16]. Several signals, including mTOR, have also been reported to be involved in fibrogenesis [27]. However, the molecular mechanisms of the anti-fibrotic potential of PA in chronic kidney fibrosis have not been elucidated.

TGF-β signaling regulates a few biological properties in cancer, including growth, apoptosis, differentiation, migration, invasion, angiogenesis, ECM production, and cancer cell interactions with the immune system [28]. Several studies have reported that TGFβ is an anti-inflammatory cytokine that plays a protective role in immune inflammation and autoimmune diseases [7,29,30]. TGF-β1 is a key mediator in renal fibrosis39, and SMAD and non-SMAD signaling is a major intracellular signaling pathway of TGF-β action in progressive renal fibrosis [31]. Recently study focus on TGF-β1 signal pathways and describe small molecule inhibitors that are used in phase I/II clinical trials to treat fibrosis or fibrotic cancers [7]. Therefore, inhibition of TGF-β1 and regulation of downstream signaling pathways play an important role in fibrosis.

In this study, we obtained the first evidence that PA inhibits obstructive nephropathy and TGF-β1-stimulated kidney fibroblast cell activation through downregulation of SMAD and non-SMAD signaling, thereby inhibiting renal fibrosis.

Extracellular matrix (ECM) deposition and epithelial-to-mesenchymal transition (EMT) progress are major causes of fibrosis in the kidney [32]. In humans with fibrotic kidneys, strong mesenchymal marker expression is accompanied by deposition of type-I collagen among the renal tubules and massive interstitial fibrosis in the renal cortex [33].

Studies of UUO mice have shown that fibroblasts and myofibroblasts, identified by the markers FSP-1 and α-SMA respectively, increase after 7 days, thus indicating EMT activation [9]. Decreased E-cadherin and increased α-SMA expression are typical EMT features [32]. In this study, PA suppressed UUO-induced tubular interstitial fibrosis by reducing the deposition of type-I collagen and increasing resistance to the expression of EMT markers.

TGF-β1 mediators progress renal fibrosis by stimulating ECM deposition and EMT [34]. It is considered the most important pathway in fibrosis and appears to be dependent on SMAD-STAT signaling [8].

Our previous studies have shown that inhibition of pSMAD has a protective effect against liver fibrosis [17]. Elsewhere, the selective inhibitor STAT3 attenuated renal fibrosis by inactivating interstitial fibroblasts in vivo [20]. In accordance with these findings, our results showed that PA effectively inhibits pSMAD3 and pSTAT3 through obstructive nephropathy. Consequently, TGF-β1-induced renal fibroblast cell activation was suppressed through the inhibition of pSMAD3 and pSTAT3 by PA.

Our findings show that PA plays a protective role against UUO-induced tubular interstitial fibrosis and against TGF-β1 induced renal fibroblast cell activation, specifically through the inhibition of the SMAD3-STAT3 signaling pathway. Based on the literature and our findings, PA should be considered a novel therapeutic agent for chronic kidney disease.

## 4. Materials and Methods

### 4.1. Cell Cultures and Reagents

NRK-49F cells (CRL1570) were obtained from America Tissue Culture Collection (ATCC, VA, USA). Cells were cultured in DMEM supplemented with 10% fetal bovine serum (FBS) and 1% antibiotic (Ab). DMEM, FBS, Ab, and trypsin-EDTA were obtained from Gibco BRL (Grand Island, NY, USA). NRK-49F cells were pretreated with PA for 1 h and then treated with TGF-β1 for 24 or 48 h. Recombinant TGF-β1 was purchased from R&D System Inc. (Minneapolis, MN, USA). Pomolic acid (PA) was purified and received from Dr. Ki Yong Lee, a professor of the College of Pharmacy, Korea University [13,15,16]. PA was dissolved in dimethylsulfoxide (DMSO) as a 10 mM stock solution and stored at 4 °C. NRK-49F cells were pretreated with PA for 1 h and then treated with TGF-β1 for 24 or 48 h.

### 4.2. Cytotoxicity Assay

Cells were plated in 96-well culture plates at 1 × 10^6^ cells/mL in culture medium and allowed to attach for 24 h. Media were then discarded and replaced with new medium containing various concentrations of PA. The cells were cultured for an additional 24 h, and then 3-(4,5-dimethylthiazol-2-yl)-2, 5-diphenylterazolium bromide (MTT, 5 mg/mL; Sigma-Aldrich, St Louis, MO, USA) was added 1/10 volume MTT reagent of medium to each well, and the samples were incubated at 37, 35 °C in a 5% CO_2_ incubator for 4 h. The formazan precipitate was dissolved in dimethyl sulfoxide (DMSO), and absorbance was measured at 540 nm using a microplate reader (Bio-Rad Laboratories, Richmond, CA, USA).

### 4.3. Induction of UUO Injury

Male BALB/c mice (Orient, Sungnam, Korea), were randomly divided into four groups of six mice per group: the group was anesthetized and underwent a similar surgical procedure of UUO but was not subjected to ureteral ligation (Sham), a Sham group with a PA treatment (PA), group underwent a vehicle treatment for UUO (UUO); and the fourth group consisted of UUO mice treated with PA (PA/UUO). We have previously reported the UUO models [1,23,35]. An intraperitoneal injection of PA at a concentration of 0.4 mg/kg was given immediately after ureteral ligation. Then, PA was given with an intraperitoneal injection 2 days after the UUO operation. The kidneys were collected for protein analyses, including a histologic examination, on day 7 post-UUO surgery. All experimental procedures used in the current study were approved by the institutional animal care and use committee at the Daegu Catholic University Medical Center (EXP-IRB number: DCIAFCR-160726-9-Y).

### 4.4. Pathology

Tissue sections were routinely fixed in 4% phosphate-buffered paraformaldehyde and paraffin embedded. Hematoxylin and eosin (H&E), Masson’s trichrome, and periodic acid–Schiff (PAS) staining were performed according to a previously described procedure [1]. The H&E staining were observed for the extent of interstitial fibrosis, tubular atrophy and interstitial inflammatory cell infiltration. Thirty glomeruli were randomly selected in the section from each kidney, and PAS-positive areas were observed. To evaluate tubulointerstitial collagen deposition, ten randomly selected fields in each section stained with Masson’s trichrome. The area stained in light blue in the interstitium was semiquantitatively calculated using i-Solution Lite V.9.1 Image Analysis Software (IMTechnology, Vancouver, BC, Canada).

For immunofluorescent staining, sections were incubated with anti-E-cadherin (#3195, Cell signaling, Danvers, MA, USA), α-SMA (#48938, Cell signaling), pSMAD3 (#9520, Cell signaling), pSTAT3 (#9145, Cell signaling) for 1 h at 37 °C, and secondary antibodies conjugated with Alexa Flour 488 (excitation/emission = 495/519 nm, green, Invitrogen, Carlsbad, CA, USA) and Alexa Flour 594 (excitation/emission = 590/617 nm, red, Invitrogen) were purchased from Invitrogen. Cells were counterstained with Hoechst 33342 (excitation/emission = 330 − 380 nm/460 nm, ImmunoChemistry, Bloomington, MN, USA). Slides were mounted using ProLong® Gold antifade reagent (Molecular Probes^®^ by Life Technologies™, Carlsbad, CA, USA). Immunolabeling was examined using an Eclipse Ti-U and confocal microscope (Nikon, Tokyo, Japan).

### 4.5. Immunoblot Analysis

The tissues and cell protein were obtained as previously described [1,17]. The protein concentration was determined with a Bio-Rad Bradford kit (Bio-Rad Laboratories, Hercules, CA, USA). The samples were boiled for 5 min, and equal volumes were loaded on a sodium dodecyl sulfate polyacrylamide gel electrophoresis. The resolved proteins were transferred onto a nitrocellulose membrane (Millipore Corporation, Bedford, MA, USA) and probed with type-I collagen (#ab34710, Abcam, Cambridge, UK), fibronectin (#sc71113, Santa Cruz, CA, USA), PAI-1 (#sc8979, Santa Cruz), E-cadherin (#3195, Cell signaling), α-SMA (#48938, Cell signaling), vimentin (#5741, Cell signaling), SMAD3 (#9523, Cell signaling), pSMAD3 (#9520, Cell signaling), STAT3 (#9139, Cell signaling), pSTAT3 (#9145, Cell signaling), and β-Actin (#4970, Cell signaling) followed by a secondary antibody conjugated to horseradish peroxidase and detected with enhanced chemiluminescence reagents (Amersham Bioscience, Buckinghamshire, UK). The luminescent signals were analyzed using an ImageQuant LAS 4000 Scanner of GE Healthcare (Piscataway, NJ, USA).

### 4.6. Statistical Analysis

All data were analyzed using ANOVA with GraphPad Prism 5 software (GraphPad Software, Inc., San Diego, CA, USA). Post hoc tests were completed with Tukey’s multiple comparison test significance set at *p* < 0.05. All values are expressed as mean ± standard error of the mean (SEM).

## Abbreviations

TGF-β1	Transforming growth factor β1
SMAD3	Mothers against decapentaplegic homolog 3
STAT3	Signal transducer and activator of transcription 3
PA	Pomolic acid
ECM	Extracellular matrix
EMT	Epithelial-mesenchymal transition
α-SMA	α-Smooth muscle actin
mTOR	Mammalian target of rapamycin
DMSO	Dimethylsulfoxide
